# SVCT2/SLC23A2 is a sodium-dependent urate transporter: functional properties and practical application

**DOI:** 10.1016/j.jbc.2023.104976

**Published:** 2023-06-28

**Authors:** Yu Toyoda, Hiroshi Miyata, Ryuichiro Shigesawa, Hirotaka Matsuo, Hiroshi Suzuki, Tappei Takada

**Affiliations:** 1 Department of Pharmacy, The University of Tokyo Hospital, Bunkyo-ku, Tokyo, Japan; 2 Department of Integrative Physiology and Bio-Nano Medicine, National Defense Medical College, Tokorozawa, Saitama, Japan

**Keywords:** uric acid, urate importer, cell-based urate efflux assay, vitamin C transporter, ascorbic acid, anion transport, ascorbate, purine, ABCG2

## Abstract

Urate transporters play a pivotal role in urate handling in the human body, but the urate transporters identified to date do not account for all known molecular processes of urate handling, suggesting the presence of latent machineries. We recently showed that a urate transporter SLC2A12 is also a physiologically important exporter of ascorbate (the main form of vitamin C in the body) that would cooperate with an ascorbate importer, sodium-dependent vitamin C transporter 2 (SVCT2). Based on the dual functions of SLC2A12 and cooperativity between SLC2A12 and SVCT2, we hypothesized that SVCT2 might be able to transport urate. To test this proposal, we conducted cell-based analyses using SVCT2-expressing mammalian cells. The results demonstrated that SVCT2 is a novel urate transporter. Vitamin C inhibited SVCT2-mediated urate transport with a half-maximal inhibitory concentration of 36.59 μM, suggesting that the urate transport activity may be sensitive to physiological ascorbate levels in blood. Similar results were obtained for mouse Svct2. Further, using SVCT2 as a sodium-dependent urate importer, we established a cell-based urate efflux assay that will be useful for identification of other novel urate exporters as well as functional characterization of nonsynonymous variants of already-identified urate exporters including ATP-binding cassette transporter G2. While more studies will be needed to elucidate the physiological impact of SVCT2-mediated urate transport, our findings deepen understanding of urate transport machineries.

Uric acid is the final metabolite in purine metabolism in humans due to a genetic loss of uricase activity (1). Accumulating evidence suggests that urate maintenance at optimal concentrations in our body is important for a healthy life, since elevated serum urate (hyperuricemia which is defined as serum urate > 7 mg/dl, 0.42 mM) is the major risk factor for gout and found to be associated with various diseases including hypertension and cardiovascular diseases (2, 3). Under physiological conditions, uric acid mainly exists as its anion form—urate, thereby it cannot passively penetrate the plasma membrane. Accordingly, urate transporters play pivotal roles in urate absorption, distribution, and excretion. Given that urate transport has been recognized as a therapeutic target for urate regulation, together with the fact that the whole picture of urate transport systems remains to be elucidated, deeper understanding of such physiologically important systems should be a significant issue.

Hitherto, we and others have identified such physiologically important urate transporters of which functional changes influence serum urate levels, in solute carrier (SLC) 22A family proteins including urate transporter 1 (URAT1)/SLC22A12 (4) and organic anion transporter 10 (OAT10)/SLC22A13 (5, 6, 7); SLC2A family proteins including glucose transporter 9 (GLUT9)/SLC2A9 (7, 8, 9) and GLUT12/SLC2A12 (10) and ATP-binding cassette (ABC) proteins such as ABC transporter G2 (ABCG2) (11, 12). These transporters account for some molecular pathways involved in urate handling, including renal urate reuptake mediated by the URAT1–GLUT9 axis (13) and intestinal urate secretion by ABCG2 (14). However, there are other urate-transporting functions for which the machineries that are responsible remain unknown, suggesting the existence of latent urate transporter proteins.

We previously identified SLC2A12 as a urate transporter, knockout of which affected serum urate levels in a mouse hyperuricemia model (10). In a more recent study (15), we further showed that SLC2A12 is also a physiologically important vitamin C exporter expressed on the apical side of choroid plexus epithelial cells, where it would be involved in the transcellular transport of vitamin C from the blood into the brain in cooperation with sodium-dependent vitamin C transporter 2 (SVCT2)/SLC23A2, a vitamin C importer expressed on the basal (blood) side of choroid plexus epithelial cells (16, 17, 18). Based on the following observations, we hypothesized an overlap in substrate specificity between SVCT2 and SLC2A12 that might allow SVCT2 to recognize urate as a substrate in addition to ascorbate: (i) vectorial transport of vitamin C (as ascorbate, a reduced form of vitamin C) mediated by SVCT2 and SLC2A12, (ii) dual functionality of SLC2A12 as a urate and ascorbate transporter, and (iii) the fact that uric acid and vitamin C are physiologically important antioxidants abundantly present in blood in monoanion forms. Moreover, SVCT2 is reportedly expressed in almost all tissues (17, 18, 19) and is localized on the basolateral membrane when expressed in polarized cells (15, 20), implying an involvement in the uptake of substrates from blood.

The aim of this study was to test our proposal that SVCT2 can transport urate, making use of *in vitro* cell-based analyses. As predicted, our results showed SVCT2 to be a sodium-dependent urate transporter. While SVCT2 is characterized as a high-affinity (low-capacity) ascorbate transporter (21), our findings showed that in urate transport it has opposite properties.

## Results

### Functional expression of SVCT2 as an ascorbate transporter

**Figure 1:**
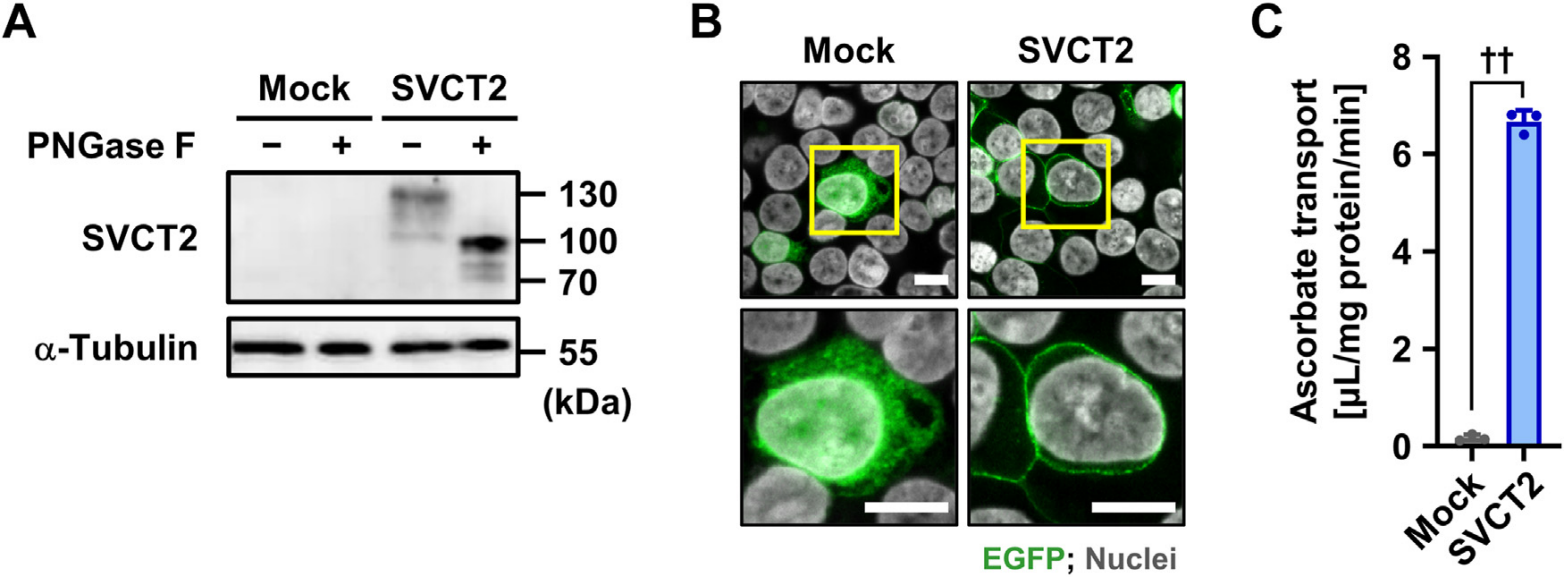
**Functional expression of SVCT2 in HEK293 cells.** Experiments used HEK293 cells transiently expressing SVCT2 48 h after plasmid transfection. *A*, immunoblot detection of SVCT2 protein in whole-cell lysates. α-Tubulin, a loading control; mock, empty vector (pEGFP-N1 plasmid without insert)-transfected control, of which results were consistent with previous studies (10, 45). *B*, intracellular localization of SVCT2 detected by confocal microscopy. Magnified images of representative cells in *yellow boxes* are shown. Scale bars, 10 μm. *C*, [1-^14^C]-ascorbate transport into cells. Data are expressed as the mean ± SD; *n* = 3. ^††^*p* < 0.01 (two-sided *t* test). SVCT2, sodium-dependent vitamin C transporter 2.

Prior to biochemical investigations for potential SVCT2 activity as a urate transporter, we verified the functional expression of SVCT2 in a cell-based assay system (Fig. 1). Expression of EGFP-tagged SVCT2 (SVCT2-EGFP) as a matured *N*-linked glycoprotein (Fig. 1*A*) and localization to the plasma membrane (Fig. 1*B*) following plasmid transfection in HEK293 cells were confirmed by immunoblotting and confocal microscopy, respectively. SVCT2-mediated uptake of ascorbate, a well-known substrate, into SVCT2-expressing cells was also confirmed (Fig. 1*C*).

### Identification and characterization of human SVCT2 as a urate transporter

**Figure 2:**
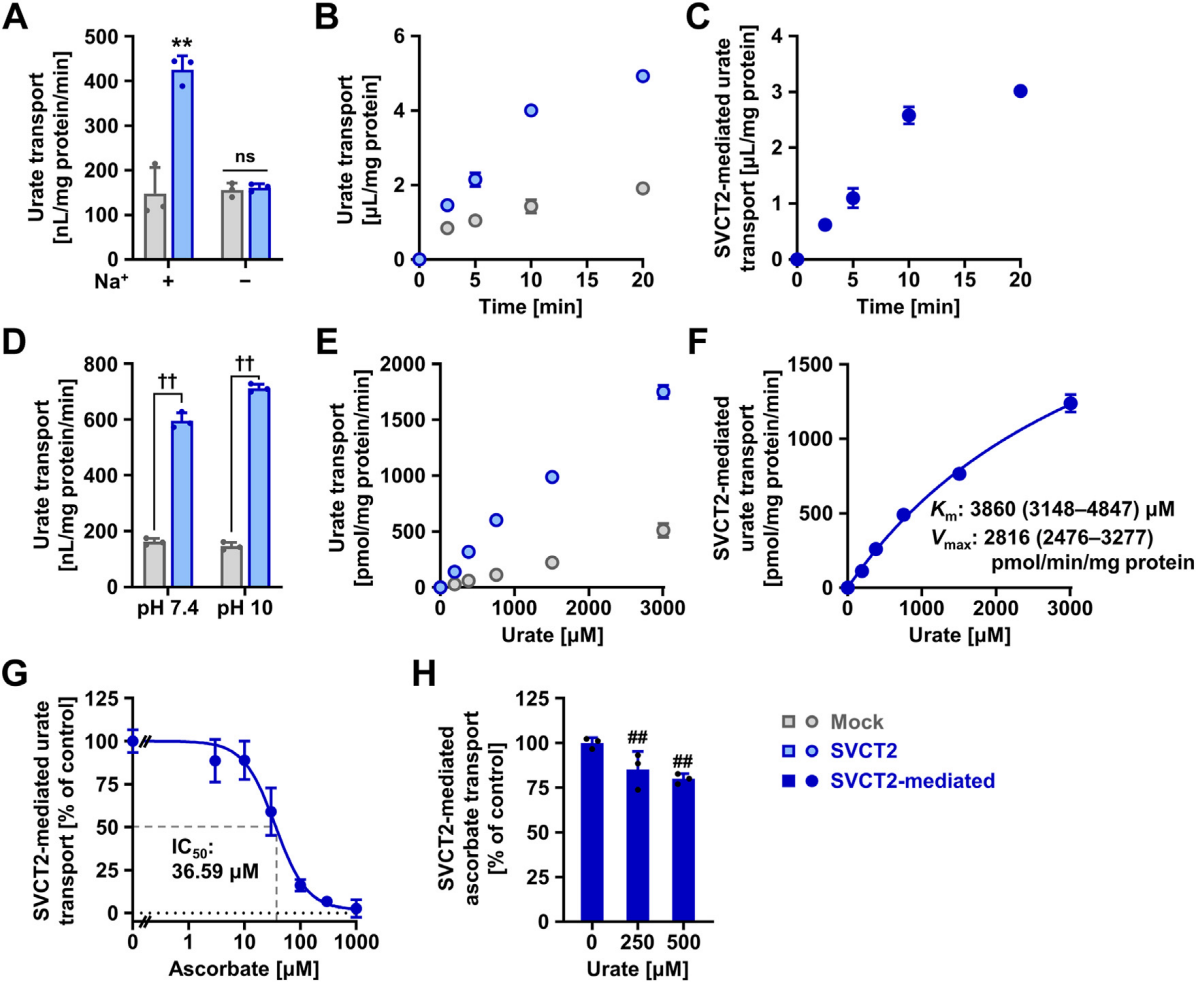
**Identification and characterization of human SVCT2 as a urate transporter.** Uptake assays were conducted in Krebs–Ringer buffer using HEK293 cells transiently expressing SVCT2 48 h after plasmid transfection. Experimental conditions were as follows: pH 7.4 (*A*–*C*, *G* and *H*), pH 10 (*E* and *F*); incubation time and [8-^14^C]-urate in the transport buffer were 5 min and 10 μM, respectively, unless otherwise specified; [1-^14^C]-ascorbate in the transport buffer was 20 μM. Mock, empty vector (pEGFP-N1 plasmid without insert)-transfected control. *A*, SVCT2 is a sodium-dependent urate transporter. *B*, time-dependent [8-^14^C]-urate uptake into SVCT2-expressing cells or mock cells. *C*, time profile for SVCT2-mediated [8-^14^C]-urate uptake into HEK293 cells, calculated by subtracting the urate transport activity of mock cells from that of SVCT2-expressing cells. *D*, urate transport activities of SVCT2 was maintained in an alkalic-pH condition (pH 10). *E*, concentration dependence of [8-^14^C]-urate uptake into SVCT2-expressing cells or mock cells. *F*, concentration dependence of SVCT2-mediated transport of [8-^14^C]-urate. For the estimated values of Michaelis–Menten constant (*K*_m_) and maximal velocity (*V*_max_), the 95% confidence interval is given in parentheses. *G*, concentration-dependent inhibition of SVCT2-mediated urate transport by ascorbate. IC_50_, the half-maximal inhibitory concentration. *H*, mild inhibitory-effect of urate on SVCT2-mediated ascorbate transport at physiological concentrations. Values are shown as % of vehicle control (*G* and *H*). Data are expressed as the mean ± SD; where the vertical bars are not visible, the SD was contained within the limits of the symbol; *n* = 3. ns, not significantly different between groups; ^∗∗^*p* < 0.01 *versus* the other groups (Tukey–Kramer multiple-comparison test); ^††^*p* < 0.01 (two-sided *t* test); ^##^*p* < 0.01 *versus* vehicle control (Williams’ multiple-comparison test). SVCT2, sodium-dependent vitamin C transporter 2.

Next, we used transiently SVCT2-expressing HEK293 cells to examine whether SVCT2 can transport urate. When cells were incubated in Krebs–Ringer buffer, [8-^14^C]-urate was taken up into SVCT2-expressing cells at higher levels than into mock cells. Exclusion of Na^+^ from Krebs–Ringer buffer abolished SVCT2-mediated urate transport (Fig. 2*A*). The results indicate that SVCT2 is a sodium-dependent urate transporter that could in principle serve as machinery for urate uptake from extracellular fluids into cells. Prior to further functional analyses, we confirmed that the EGFP tag had little effect on the cellular function of SVCT2 as a urate transporter (Fig. S1).

To gain more insight into the biochemical features of SVCT2 as a urate transporter, we conducted further analyses. SVCT2-mediated urate uptake increased linearly with time for at least 10 min (Fig. 2, *B* and *C*), which allowed us to use urate uptake at 5 min for the evaluation of initial rates of SVCT2-mediated transport in subsequent analyses. Given the low-solubility limitation of uric acid under neutral conditions, in order to achieve high urate concentrations in the uptake assay, we employed high pH conditions for kinetic analyses (Krebs–Ringer buffer at pH 10), after first showing that the change in pH had little effect on SVCT2-mediated urate transport (Fig. 2*D*). In the urate concentration range tested (0–3 mM, approximately 50 mg/dl at the high end), SVCT2-mediated urate transport was not saturated (Fig. 2, *E* and *F*), suggesting that SVCT2 mediated robust transport of urate. The parameters calculated for SVCT2-mediated urate transport were Michaelis–Menten constant (*K*_m_) 3.86 mM and maximal velocity (*V*_max_) 2816 pmol/min/mg protein (Fig. 2*F*); R square was 0.996. Given the range of physiological concentrations for serum urate in healthy humans [120–420 μM (approximately 2–7 mg/dl) (22, 23)], SVCT2 should be able to maintain urate transport activity from the viewpoint of the substrate concentration in our body.

As ascorbate is a physiologically relevant substrate of SVCT2, we investigated its effect on SVCT2-mediated urate transport at pH 7.4 (Fig. 2*G*). The results showed concentration-dependent inhibition with a half-maximal inhibitory concentration (IC_50_) value of 36.59 μM. From a previous study (24), steady-state plasma vitamin C concentrations are approximately 50 to 90 μM in healthy subjects with ≥100 mg of daily vitamin C intake, corresponding roughly to the recommended dietary allowance (the average daily intake sufficient to meet the nutrient requirements of 97–98% of healthy individuals) of 90 mg in adult (≥19-year-old) men (25). Based on this information, SVCT2-mediated urate transport from blood into tissues or organs may be affected by circulating vitamin C, while transport would not be completely inhibited. We also found that at the physiological pH, urate inhibited SVCT2-mediated ascorbate transport, although the inhibitory effect was not so large, with inhibition by 500 μM urate amounting to only 20% (Fig. 2*H*).

### Identification and characterization of mouse Svct2 as a urate transporter

**Figure 3:**
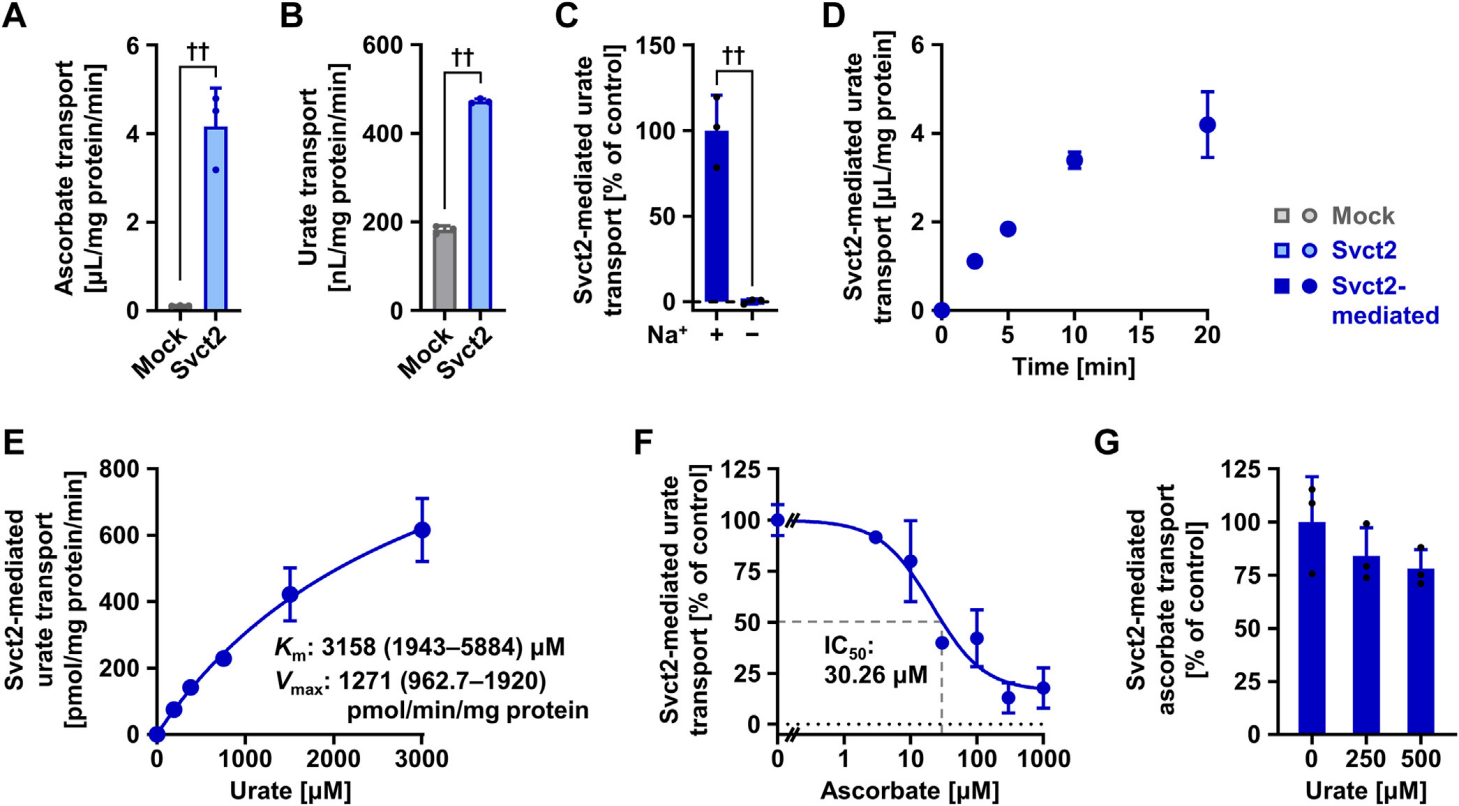
**Identification and characterization of mouse Svct2 as a urate transporter.** Uptake assays were conducted in Krebs–Ringer buffer using HEK293 cells transiently expressing Svct2 48 h after plasmid transfection. Experimental conditions were as follows: pH 7.4 (*A*–*D*, *F*, and *G*), pH 10 (*E*); 20 μM [1-^14^C]-ascorbate in the transport buffer (*A* and *G*); incubation time and [8-^14^C]-urate in the transport buffer were 5 min and 10 μM, respectively, unless otherwise specified. Mock, empty vector (pEGFP-N1 plasmid without insert)-transfected control. *A*, [1-^14^C]-ascorbate uptake into SVCT2-expressing cells or mock cells. *B*, [8-^14^C]-urate uptake into SVCT2-expressing cells or mock cells. *C*, sodium-dependent urate transport mediated by Svct2. *D*, time profile for Svct2-mediated [8-^14^C]-urate uptake into HEK293 cells. *E*, concentration dependence of Svct2-mediated transport of [8-^14^C]-urate. For the estimated Michaelis–Menten constant (*K*_m_) and maximal velocity (*V*_max_), the 95% confidence interval is given in parentheses. *F*, concentration-dependent inhibition of Svct2-mediated urate transport by ascorbate. IC_50_, the half-maximal inhibitory concentration. *G*, mild inhibitory-effect of urate on Svct2-mediated ascorbate transport at physiological concentrations. Values are shown as % of vehicle control (*F* and *G*). Data are expressed as the mean ± SD; where the vertical bars are not visible, the SD was contained within the limits of the symbol; *n* = 3. ^††^*p* < 0.01 (two-sided *t* test); no significant differences among groups (Williams’ multiple-comparison test) were found (*G*). SVCT2, sodium-dependent vitamin C transporter 2.

Subsequently, we studied mouse Svct2 and obtained similar results (Fig. 3). When expressed in HEK293 cells, Svct2 mediated both ascorbate (Fig. 3*A*) and urate (Fig. 3*B*) uptake into the cells; the EGFP tag had little effect on the cellular function of Svct2 as a urate transporter (Fig. S2). Svct2-mediated urate transport was sodium-dependent (Fig. 3*C*) and increased in a linear fashion during the first 10 min (Fig. 3*D*). Based on the time-course experiment, we investigated concentration-dependent urate uptake at 5 min to determine the kinetic parameters. As for SVCT2 (Fig. 2*F*), Svct2-mediated urate transport was not saturated in the range examined (0–3 mM) (Fig. 3*E*). The calculated parameters for Svct2-mediated urate transport were *K*_m_ 3.16 mM and *V*_max_ 1271 pmol/min/mg protein; R square was 0.997. The results suggest that Svct2 also has a robust function for urate transport and that there should be little difference in substrate specificity to urate between human SVCT2 and mouse Svct2. Further analyses focusing on the interaction between the Svct2 substrates urate and ascorbate showed that ascorbate inhibited Svct2-mediated urate transport with an IC_50_ value of 30.26 μM (Fig. 3*F*). On the other hand, while there was a slight declining trend as a function of concentration, no significant inhibition of Svct2-mediated ascorbate transport by urate was observed at physiological concentrations (Fig. 3*G*). Overall, the results were consistent with those for human SVCT2.

### Establishment of a cell-based urate efflux assay

**Figure 4:**
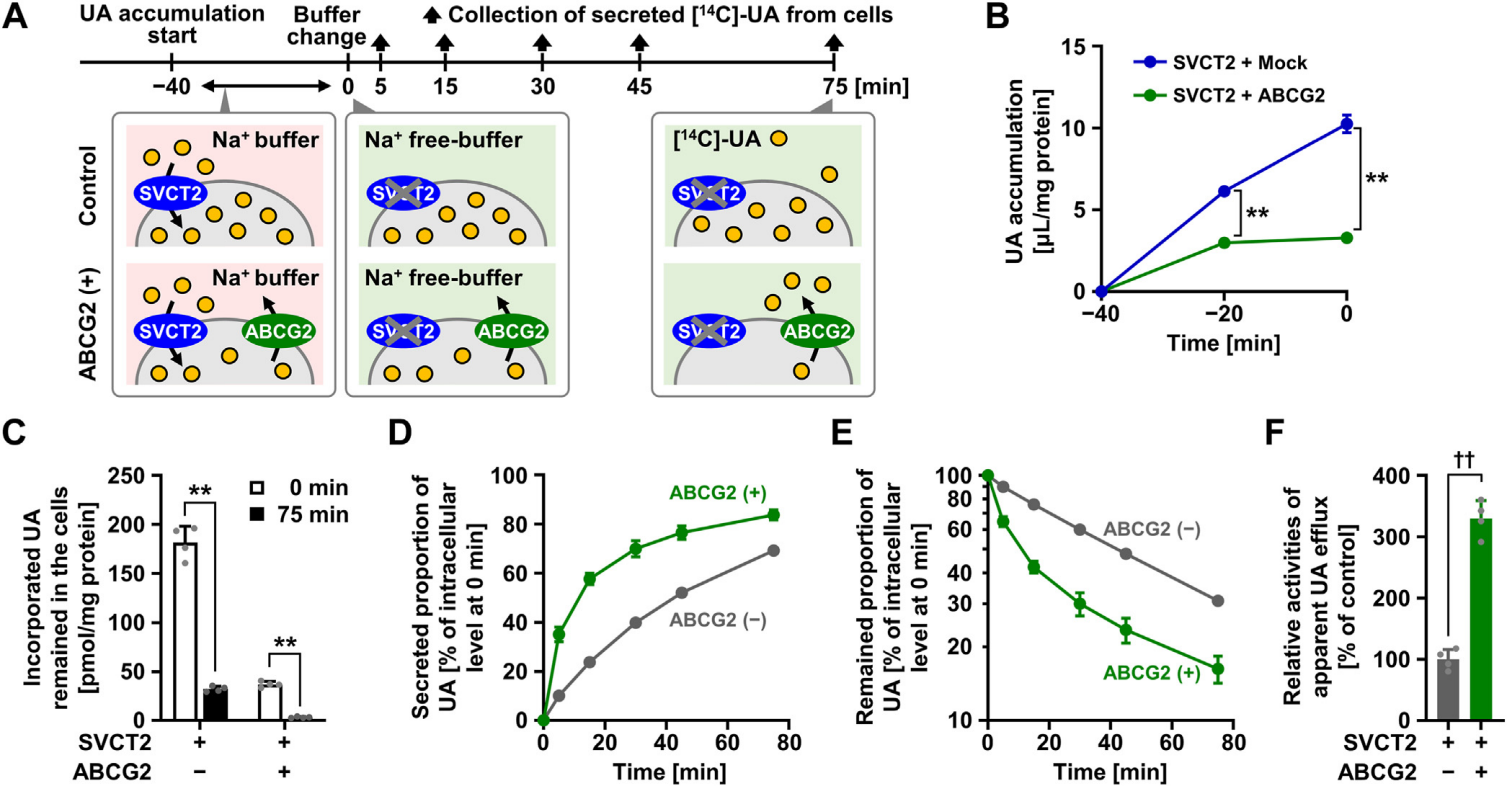
**Establishment of a cell-based urate efflux assay.** Experiments used HEK293-derived 293A cells transiently double-expressing SVCT2 and ABCG2 48 h after plasmid transfection. UA, urate. Mock, empty vector (pEGFP-C1 plasmid without insert)-transfected control corresponding to ABCG2 (−) condition. *A*, schematic illustration of [8-^14^C]-urate efflux assay. *B*, SVCT2-mediated [8-^14^C]-urate accumulation in cells prior to the efflux assay. *C*, [8-^14^C]-urate levels in cells at the start and end of the efflux phase (0–75 min). *D*, time-dependent increase in secreted [8-^14^C]-urate from cells with or without ABCG2 expression. Urate efflux was evaluated as secreted proportion that was defined as the ratio of media-released amount of [8-^14^C]-urate against its cellular amount at 0 min. *E*, time-dependent decrease in cellular [8-^14^C]-urate. Each value of remained proportion was calculated by subtracting the corresponding value of secreted proportion at indicated time point from 100 [%]. A base-10 logarithmic scale was employed for the vertical axis. *F,* comparison of apparent urate efflux activities between ABCG2-expressing cells and control cells. The activities were expressed as a percentage of control. Data are expressed as the mean ± SD; where the vertical bars are not visible, the SD was contained within the limits of the symbol; *n* = 3 (*B*), 4 (*C*–*F*). ^∗∗^*p* < 0.01 (Scheffe’s *F* test among all the groups in each panel); ^††^*p* < 0.01 (two-sided *t* test). ABCG2, ATP-binding cassette transporter G2; SVCT2, sodium-dependent vitamin C transporter 2.

Finally, using the sodium dependency of the SVCT2-mediated urate transport, we established a cell-based urate efflux system comprising two steps (Fig. 4*A*). The first step is urate accumulation in which extracellular [8-^14^C]-urate is taken up into cells *via* overexpressed SVCT2. The second step is carrier-mediated urate efflux, whereby [8-^14^C]-urate taken up in the first step is secreted into a medium that should be sodium free, a condition that deactivates SVCT2-mediated urate uptake during the efflux assay. For a proof of concept, we herein chose ABCG2, a physiologically important urate exporter localized on the plasma membrane (11, 12), as a partner to SVCT2 in the efflux system. We used human HEK293-derived 293A cells as the host cell line because of high transfection efficiency and lack of uricase, a urate-degrading enzyme not existing in humans (1).

In the accumulation step, although co-transduction of ABCG2 decreased the apparent urate uptake activity mediated by SVCT2, [8-^14^C]-urate accumulated in cells incubated in Krebs–Ringer buffer during 40 min (Fig. 4*B*). In the secretion step, regardless of the presence or absence of ABCG2 co-expression, cellular [8-^14^C]-urate levels at the end of the assay (75 min) were significantly lower than at the start (0 min) (Fig. 4*C*). These results indicated that cellular activities for urate secretion were higher than those for urate uptake in both groups under the sodium-free condition. Also, at the start of secretion step (*t* = 0 min), cellular levels of [^14^C]-urate in ABCG2-expressing group were lower than those in control group that had no transient expression of ABCG2. This result shows that even during the urate accumulation step, ABCG2-mediated urate efflux from cells partially cancelled out the SVCT2-mediated urate uptake into the cells, resulting in the decrease in the net amount of accumulated [^14^C]-urate. This difference in baseline would not allow us to evaluate the apparent cellular activity for urate secretion as its efflux rate (*i.e*., pmol/min in this study). Instead, we used normalized values based on the intracellular levels of [^14^C]-urate, according to the clearance concept.

Considering the above, we addressed the effect of ABCG2 transduction on the cellular efflux of urate. First, we simply evaluated the efflux as secreted proportion to the initial cellular amount (net amount of media-released [8-^14^C]-urate/initial amount of cellular [8-^14^C]-urate at 0 min) (Fig. 4*D*). As expected, time-dependent change in the proportion in ABCG2-expressing cells was greater than in control cells. The corresponding decrease in cellular [8-^14^C]-urate was shown in Fig. 4*E* with a base-10 logarithmic scale of the vertical axis. Based on these results, we decided to calculate an apparent urate efflux activity (details are shown in Experimental procedures) as a trend over 0 to 5 min; the activities in ABCG2-expressing cells were significantly higher (approximately 3.3-fold) than those in control cells (Fig. 4*F*). Importantly, ABCG2 is a high-capacity urate transporter of which *K*_m_ value for urate is 8.2 mM (12); this means that ABCG2 maintains its cellular function even under high-urate conditions, suggesting that the cellular ABCG2 must have not been saturated during the efflux step. Given the successful detection of accelerated urate secretion from ABCG2-expressing cells, the cell-based assay would be useful for evaluation of urate efflux activities of other (latent) urate exporters.

## Discussion

In this study, we successfully identified human SVCT2 (Fig. 2) and mouse Svct2 (Fig. 3) as sodium-dependent robust urate importers. Given their role in facilitating accumulation of vitamin C as ascorbate in many tissues (17), and the fact that under physiological conditions uric acid and vitamin C predominantly exist as monoanionic urate and ascorbate, respectively, SVCT2 may be involved in urate uptake from blood into the tissues. However, its physiological impact on urate handling should be verified by further investigations. Focusing on the sodium dependency in the SVCT2-mediated urate uptake, we established a cell-based urate efflux assay (Fig. 4) that would be useful for functional validation of urate exporters localized on the plasma membrane.

We revealed that SVCT2 is an ascorbate and urate transporter; however, the characteristics of SVCT2-mediated transport are different between the substrates. As summarized in the literature (21), previous studies have shown that SVCT2 in humans or rodents is a high-affinity (low-capacity) ascorbate transporter with *K*_m_ values for ascorbate of 10 to 100 μM. In contrast, our results showed that SVCT2 has a low affinity for urate with a *K*_m_ for urate of 3.86 mM (Fig. 2*F*). Given that the *K*_m_ value is larger than those of previously characterized physiologically important urate importers [371 μM, URAT1/SLC22A12 (4); 558 μM, OAT10/SLC22A13 (5)], SVCT2 can be categorized as a low-affinity urate transporter, similar to ABCG2 with a *K*_m_ for urate of 8.3 mM (12). Regarding the substrate specificity of SVCT2, its higher affinity for ascorbate rather for urate is reasonable, given its physiological role in vitamin C transport into many tissues, together with the physiological concentrations of ascorbate (50–90 μM) and urate (120–420 μM) in the blood of healthy humans. In other words, these kinetic features allow SVCT2 to successfully transport ascorbate from blood in the presence of urate, consistent with our *in vitro* results showing only a small impact of urate on SVCT2-mediated ascorbate transport (Fig. 2*H*).

There are further biological implications of this dual functionality of SVCT2, even though its physiological role as a urate transporter remains unclear. One plausible scenario is that SVCT2 may be involved in cellular uptake of urate as an alternative antioxidant to ascorbate when there is a deficiency of vitamin C. Given the potential inhibitory effects of ascorbate on SVCT2-mediated urate transport *in vivo* suggested by this study (Fig. 2*G*), SVCT2-mediated urate transport activities could increase several-fold if ascorbate concentrations were greatly decreased in humans, a species that is unable to synthesize vitamin C. Addressing this possibility of compensatory activation *in vivo* will be a matter for future studies and may uncover latent interactions between urate and ascorbate.

Our cell-based urate efflux assay should represent a convenient system for functional characterization of ABCG2 variants. Recent clinicogenetic studies have revealed the clinical importance of dysfunctional variations of *ABCG2* as genetic risk factors for such urate-related diseases including gout and hyperuricemia (11, 12, 26, 27, 28) and have attracted interest in functional validation of non-synonymous ABCG2 variants even though the allele frequency is extremely low (29, 30, 31). To date, determining the effects of amino acid substitutions on ABCG2 function as a urate exporter has relied on *in vitro* vesicle transport assays (32), which require preparation of ABCG2-expressing plasma membrane vesicles from ABCG2-overexpressing cells and a subsequent transport assay using the vesicles. While the vesicle transport assay has the advantage of detecting direct transport of urate mediated by ABCG2 in an ATP-dependent manner, the multistep nature of the assay offers disadvantage in addressing multiple variants in the same lot of experiment. The cell-based urate efflux assay established here offers a solution. Moreover, the cell-based assay can be applied to identify orphan transporters for which the driving force remains unclear, to examine whether candidates possess urate efflux activity.

Some limitations and future directions of the study warrant mention. First, although we identified SVCT2 as a urate transporter, its physiological role in urate handling remains unclear. Although little information is available on molecular mechanisms regulating the cerebral distribution of urate, a previous study expected that latent machineries may be involved in the uptake of urate from blood into choroid plexus epithelial cells (33). Given the results of this study, together with the expression of SVCT2 on the basal side of the cells, whether SVCT2 has such a role or not will be an important issue for future works. Considering that SVCT2 is a physiologically important ascorbate transporter, a deficiency of which is reportedly lethal in newborn mice (17), *in vivo* approaches based on conventional knockout of *Svct2* in mice would be difficult. However, conditional knockout focusing on particular tissues or organs may represent a path for further investigations.

Second, the low-solubility limitation of uric acid under neutral conditions could not allow us to fully investigate the concentration-dependent inhibitory effects of urate on SVCT2-mediated ascorbate transport (Fig. 2*H*), contrary to the inhibitory effects of urate on SVCT2-mediated ascorbate transport (Fig. 2*G*). However, our data suggest that urate would not have much influence on SVCT2-mediated ascorbate transport under physiological conditions, which is consistent with the fact that SVCT2 has a high affinity for ascorbate than urate as described above. Moreover, given the goodness in curve fitting of SVCT2-mediated urate transport in the Michaelis–Menten model (Fig. 2*F*; R square, 0.996), a simple single-binding site model might be applied for the urate transport. In this context, since urate and ascorbate are SVCT2 substrates, we can envision that ascorbate could competitively inhibit the urate transport. Future studies are required to address these proposals, which will deepen our understanding of the latent molecular basis on the membrane transport mediated by SVCT2 as well as mutual interaction between its substrates.

Third, [8-^14^C]-urate was also secreted from control cells in the cell-based urate efflux assay, indicating the presence of endogenous urate exporter(s) in the HEK293-derived 293A cells used, and these alternate transporters remain to be identified. Exclusion of such endogenous factors from the host cells will contribute to improving the assay system, and their molecular identification is a matter for future work.

Before closing, we would like to discuss about the candidates involved in the non-ABCG2-mediated urate efflux. Human transporters consist of two types of proteins: ABC transporters (34), of which almost all the members work as efflux machineries, and SLC transporters (35), which can be involved in bidirectional transport of substrates if conditions permit. Thus, among both types of transporter proteins, what localized on the plasma membrane in our experimental system should be the candidates for the latent urate exporter. To explore such machineries, we considered that proteome information on HEK293 cell lineage can be a clue, though cellular proteome profiles are influenced by culture conditions. Regarding this point, we addressed a public dataset registered in Expression Atlas (https://www.ebi.ac.uk/gxa/experiments/E-PROT-20/Results). According to the data obtained *via* proteomic analyses conducted in a previous study (36), 7513 proteins found in HEK293 cells contained 15 ABC transporters and 96 SLC transporters. Among them, there are no already-identified urate transporters including GLUT9, with an exception of ABCC4 of which urate transport activity was found *in vitro* (37). However, given that our previous studies demonstrated that there was little activity for ATP-dependent urate transport in plasma membrane vesicles derived from control vector-transfected 293A cells (30, 38) as well as HEK293 cells (12), endogenous ABC transporters might have hardly contributed to the non-ABCG2-mediated urate efflux detected in our assay system. Hence, focusing on the listed SLC transporters may be important to identify the latent urate transporter in future.

In summary, we showed that SVCT2 is not only an ascorbate transporter but also a urate transporter. Moreover, focusing on the sodium dependency of its transport activity, we established a convenient cell-based urate efflux assay using nonpolarized mammalian cells that are easily transfected. This assay will be useful for functional characterization of variants of urate exporters including ABCG2 as well as for exploration of latent urate exporters. While the physiological role of SVCT2 as a urate transporter remains to be investigated, our findings deepen the molecular understanding of urate handling machineries and provide a practical tool for future research on cellular urate secretion.

## Experimental procedures

### Materials

Critical materials and resources used in this study are summarized in Table 1. All other chemicals used were commercially available and of analytical grade.

**Table 1:** Key resources

Reagent or resource	Source	Identifier
Antibodies		
Rabbit polyclonal anti-EGFP	Life Technologies	Cat# A11122; RRID: AB_221569; 1:1000 dilution
Rabbit polyclonal anti-α-tubulin	Abcam	Cat# ab15246; RRID: AB_301787; 1:1000 dilution
Donkey anti-rabbit IgG-horseradish peroxidase (HRP)-conjugate	GE Healthcare	Cat# NA934V; RRID: AB_772206; 1:3000 dilution
Chemicals		
[8-^14^C]-Uric acid (53 mCi/mmol)	American Radiolabeled Chemicals	Cat# ARC0513
Uric acid	FUJIFILM Wako Pure Chemical	Cat# 210–00225; CAS: 69–93–2
Ascorbic acid, L-[1-^14^C]-(Vitamin C)	PerkinElmer	Cat# NEC146
L(+)-Ascorbic acid	FUJIFILM Wako Pure Chemical	Cat# 012–04802; CAS: 50–81–7
Polyethylenimine “MAX” (PEI-MAX)	Polysciences	Cat# 24765; CAS: 49,553–93–7
Critical Commercial Assays		
Pierce BCA Protein Assay Reagent A & B	Thermo Fisher Scientific	Cat# 23223, Cat# 23224
Recombinant DNA		
The complete human SVCT2 cDNA	Miyata *et al.* (2022) (15)	NCBI: NM_005116
The complete mouse Svct2 cDNA	Miyata *et al.* (2022) (15)	NCBI: NM_018824
The complete human ABCG2 cDNA	Takada *et al.* (2005) (40)	NCBI: NM_004827
Experimental Models: Cell Lines		
Human: HEK293 cells	Toyoda et al. (2016) (43)	N/A
Human: 293A cells	Invitrogen	R70507
Software and Algorithms		
Excel 2019	Microsoft	https://products.office.com/ja-jp/home
Statcel4 add-in software	OMS Publishing	http://www.oms-publ.co.jp/
GraphPad Prism 8	GraphPad Software	https://www.graphpad.com/

### Plasmid construction

The full-length of the wildtype (WT) human SVCT2/SLC23A2 open reading frame (ORF) (NCBI accession no. NM_005116, encoding a 650-amino acid protein) and mouse Svct2/Slc23a2 ORF (NCBI accession no. NM_018824, encoding a 648-amino acid protein) in pEGFP-N1 vector (Clontech Laboratories) were constructed in a previous study (15). Since another study reported that SVCT2-GFP, a similar construct, maintained molecular properties of SVCT2 as an ascorbate transporter (39), the EGFP tag was added to the C terminus of SVCT2 and Svct2 in this study. To restore the original termination codon of SVCT2 for the expression of SVCT2 without EGFP tag, the site-directed mutagenesis technique was successfully employed. The resulting construct (nontagged SVCT2 in pEGFP-N1) was confirmed by full sequencing using BigDye Terminator v3.1 (Applied Biosystems) on an Applied Biosystems 3130 Genetic Analyzer (Applied Biosystems). The expression vector for nontagged Svct2 in pEGFP-N1 was prepared in a similar manner.

ABCG2 WT ORF (NCBI accession no. NM_004827, encoding a 655-amino acid protein) in pEGFP-C1 vector (Clontech Laboratories) was derived from another previous study (40). Of note, literature data show that EGFP-ABCG2 is suitable for functional characterization for ABCG2 as a urate transporter (30, 38), which is consistent with other previous studies demonstrating that large fluorescent protein tags such as GFP and YFP are well tolerated in cellular processing and function of ABCG2 (41, 42).

### Cell culture

Human embryonic kidney-derived HEK293 cells were maintained in Dulbecco’s Modified Eagle’s Medium (Nacalai Tesque) supplemented with 10% fetal bovine serum (Biowest), 1% penicillin-streptomycin (Nacalai Tesque), 2 mM L-glutamine (Nacalai Tesque), and MEM nonessential amino acids (Life Technologies) at 37 °C in a humidified atmosphere of 5% (v/v) CO_2_ in air as described previously (43). Also, HEK293-derived 293A cells, which were only used for the cell-based urate efflux assay, were maintained similarly.

Vector plasmids encoding each transporter or mock vectors containing no insert (*i.e.*, empty pEGFP-N1 vector as a control for SVCT2- or Svct2-EGFP expression; empty pEGFP-C1 vector as a control for EGFP-ABCG2 expression) were transfected into HEK293 cells using polyethyleneimine “MAX” (PEI-MAX) (1 mg/ml in milliQ water, pH 7.0; Polysciences) as described previously (15, 30), with some modifications. In brief, HEK293 cells were seeded onto 12-well cell culture plates for immunoblotting and cell-based transport assays at 0.92 × 10^5^ cells/cm^2^. Twenty-four hours after seeding, cells were transiently transfected with plasmid vectors using PEI-MAX (5 μl of PEI-MAX solution/1 μg of plasmid). For immunoblotting and cell-based uptake assay, 0.75 μg of plasmid/well of 12-well cell culture plate (culture volume, 1 ml/well) was used for transient transfection. Regarding confocal microscopy and cell-based urate efflux assay, details are shown in each corresponding section. The medium was replaced with fresh medium after the first 24 h of incubation.

### Preparation of whole-cell lysates and immunoblotting

Forty-eight hours after the plasmid transfection, whole-cell lysate (WCL) samples were prepared as described previously (38). In brief, after washing twice with ice-cold phosphate-buffered saline without potassium [PBS (−)], HEK293 cells were lysed with an ice-cold RIPA lysis buffer [50 mM Tris-HCl, 150 mM NaCl, 0.1% SDS, 0.5% sodium deoxycholate, 1% NP-40, 1 mM phenylmethylsulfonyl fluoride, pH 7.4 with Protease Inhibitor Cocktail for General Use (Nacalai Tesque) at the recommended dose]. The solution was centrifuged at 15,000*g* at 4 °C for 10 min, and the resulting supernatant (WCL) was transferred to a fresh tube. The protein concentration was quantified using a Pierce BCA Protein Assay Kit (Thermo Fisher Scientific) with BSA as a standard, according to the manufacturer’s protocol. For glycosidase treatment, WCL samples were incubated with PNGase F (1.25 U/μg of protein; New England Biolabs) at 37 °C for 10 min as described previously (44), then subjected to immunoblotting.

Immunoblot analyses were performed as described previously (15). Briefly, WCL samples were separated by SDS-PAGE and transferred to an Immobilon-P PVDF membrane (Merk Millipore) by electroblotting at 15 V for 60 min. For blocking, the membrane was incubated in Tris-buffered saline containing 0.05% Tween 20 and 3% BSA (TBST-3% BSA). Blots were probed with appropriate antibodies (Table 1) in TBST-0.1% BSA for 1 h, and then an HRP-dependent luminescence was developed with ECL Prime Western Blotting Detection Reagent (GE Healthcare). Immunocomplexes were detected using a multi-imaging Analyzer Fusion Solo 4 system (Vilber Lourmat).

### Confocal microscopy

For confocal laser-scanning microscopy, specimens were prepared as described previously (10, 45), with minor modifications. Briefly, HEK293 cells were seeded onto gelatin-coated glass-bottom dishes (Matsunami Glass) at 6 × 10^5^ cells/dish. Twenty-four hours after seeding, cells were transiently transfected with 2 μg of plasmid/dish (culture volume, 2 ml/dish) as described above; 48 h after transfection, cells were fixed with ice-cold methanol. After washing with PBS (−), cells were treated with Hoechst 33342 (Thermo Fisher Scientific) for 10 min at room temperature in the dark. After visualization of the nuclei, cells were washed with PBS (−) and mounted on Fluorescence Mounting Medium (Agilent Technologies). To analyze the localization of EGFP-fused transporter proteins, fluorescence was observed using a FV10i Confocal Laser Scanning Microscope (Olympus).

### Cell-based uptake assay

To examine urate transport by human SVCT2 and mouse Svct2, cell-based urate uptake assays using human SVCT2-or mouse Svct2-expressing HEK293 cells were conducted as described in our previous studies (10, 15, 46), with some modifications. In brief, 48 h after plasmid transfection, cells were washed twice with Krebs–Ringer buffer (133 mM NaCl, 4.93 mM KCl, 1.23 mM MgSO_4_, 0.85 mM CaCl_2_, 20 mM CAPS, 5 mM D-glucose, 5 mM L-glutamine, pH 7.4 unless otherwise indicated; when using a Na^+^-free condition, NaCl was replaced with equimolar choline chloride) and were preincubated in the same buffer at 37 °C for 15 min. The buffer was replaced with prewarmed fresh buffer containing [8-^14^C]-uric acid (53 mCi/mmol; American Radiolabeled Chemicals) at 10 μM or the concentration indicated, and cells were incubated further for 10 min or the period indicated. To test ascorbate transport, [1-^14^C]-vitamin C (L-ascorbic acid) (7.3 mCi/mmol; PerkinElmer) was used at 20 μM as a substrate. After the incubation was complete, cells were washed twice with ice-cold Krebs–Ringer buffer and then lysed with 500 μl of 0.2 M NaOH on ice with gentle shaking for 1 h. Lysates were neutralized with 100 μl of 1 M HCl. Radioactivity in the lysate was measured using a liquid scintillator (Tri-Carb 3110TR; PerkinElmer). Protein concentrations in the lysates were determined using the Pierce BCA Protein Assay Kit, as described above.

Urate transport activity was calculated as incorporated clearance (μL/mg protein/min) = (incorporated level of urate [DPM/mg protein/min]/urate level in the incubation mixture [DPM/μl]). Human SVCT2- and mouse Svct2-mediated urate transport activities were calculated by subtracting the urate transport activity of mock cells from that of human SVCT2-and mouse Svct2-expressing cells, respectively. The ascorbate transport activity was calculated in a similar manner.

To determine the kinetic parameter (*K*_m_ and *V*_max_) for SVCT2-or Svct2-mediated urate transport, the Michaelis–Menten model was fitted to the experimental transport rates and urate concentrations using nonlinear regression curve fitting with GraphPad Prism 8 (GraphPad Software).

SVCT2- or Svct2-mediated urate transport activity, expressed as a percentage of control (100%), were investigated in the presence of varying concentrations of ascorbate to determine the IC_50_ value. Fitting curves were obtained according to the following formula using a least-squares method in Excel 2019 (Microsoft):

Predictedvalue[%]=100−(Emax×CnEC50n+Cn)

E_max_, the maximum effect; C, the concentration of ascorbate; EC_50_, the half maximal effective concentration; n, the sigmoid-fit factor; and IC_50_ values were calculated as previously described (44, 46).

### Cell-based urate efflux assay

As a potential application of SVCT2-mediated urate transport, we constructed an *in vitro* cell-based assay to investigate urate efflux activities of urate exporters. For this purpose, we chose ABCG2 as a urate exporter. As in our previous study that established a cell-based vitamin C efflux assay (15), we employed SVCT2, the activity of which is regulated by Na^+^ in the transport buffer (Krebs–Ringer buffer) to achieve sufficient urate uptake into cells. The cell-based urate efflux assay using SVCT2-expressing 293A cells with co-expression of ABCG2 was conducted in 12-well cell culture plates. To equalize the amount of plasmid used for transient transfection among the wells, a mock vector was used. The details are as follows.

First, for the accumulation step, 48 h after double plasmid transfection (total 1 μg: 0.5 μg of SVCT2/pEGFP-N1 and 0.5 μg of ABCG2/pEGFP-C1 or empty pEGFP-C1), cells were washed twice with Krebs–Ringer buffer and preincubated in the Krebs–Ringer buffer at 37 °C for 15 min. The buffer was then replaced with prewarmed fresh Krebs–Ringer buffer containing 10 μM [8-^14^C]-urate, and the cells were incubated further at 37 °C for 40 min to incorporate radiolabeled urate.

Next, for the secretion step, the cells were washed twice on ice with ice-cold Na^+^-free Krebs–Ringer buffer containing 5 μM nonradiolabeled urate to remove the remaining extracellular radiolabeled urate. The buffer was then replaced with 500 μl of the prewarmed Na^+^-free Krebs–Ringer buffer (time: 0 min), and the cells were incubated further at 37 °C for 75 min. At indicated time points, 100 μl of the incubation buffer was collected, and the same volume of fresh buffer was added to maintain the total volume of incubation buffer in the well.

Following incubation, the cells were washed twice with ice-cold Na^+^-free Krebs–Ringer buffer and then lysed and neutralized as described above. Radioactivity in the lysate and collected buffer samples (at 5, 15, 30, 45, and 75 min) was measured using a liquid scintillator. Protein concentrations in the lysates were determined using the Pierce BCA Protein Assay Kit as described above.

In this study, we quantitatively evaluated the cellular urate efflux as secreted proportion and apparent urate efflux activity. The former indicator was defined as follows: secreted proportion [%] = net amount of media-released [8-^14^C]-urate per well [DPM]/initial amount of cellular [8-^14^C]-urate at 0 min per well [DPM] × 100. To calculate the initial amount of cellular [8-^14^C]-urate at 0 min (which is consistent with the net amount of incorporated [8-^14^C]-urate during the accumulation step), the total amount of media-released radioactivity and intracellular radioactivity at 75 min were summed. The latter indicator was defined as follows: apparent urate efflux activity [mg protein/min] = [8-^14^C]-urate efflux rate (0–5 min) [DPM/min]/cellular [8-^14^C]-urate level at 0 min [DPM/mg protein]; the activity was expressed as a percentage of control.

### Statistics

All statistical analyses were performed using Excel 2019 with Statcel4 add-in software (OMS publishing). Different statistical tests were used for different experiments, as described in figure legends. When analyzing multiple groups, the similarity of variance among groups was compared using Bartlett’s test. When passing the test for homogeneity of variance, a parametric Tukey–Kramer multiple-comparison test for all pairwise comparisons, a Dunnett’s test for comparisons with a control group, or a parametric Williams’ multiple-comparison test for trend analysis was used; otherwise, a two-factor factorial ANOVA followed by Scheffe’s *F* test for multiple-comparison was employed. In the case of a single pair of quantitative data, after comparing the variances of a set of data using an *F*-test, an unpaired Student’s *t* test or Welch’s *t* test was performed. Statistical significance was defined in terms of *p* values less than 0.05 or 0.01.

No specific statistical test was used to predetermine the sample sizes that were empirically determined in this study. All experiments were monitored in a nonblinded fashion. Samples that had undergone technical failure during processing were excluded from analyses. The numbers of biological replicates (*n*) are given in the figure legends.

## Data availability

Data are available from the corresponding author upon reasonable request. All data relevant to the study are included in the article or uploaded as online supporting information.

## Supporting information

This article contains supporting information.

## Conflict of interest

The authors declare that they have no conflicts of interest with the contents of this article.
