# Supplementary material for: SVCT2/SLC23A2 is a sodium-dependent urate transporter: functional properties and practical application
Source: J Biol Chem. 2023 Jun 28;299(8):104976. doi: 10.1016/j.jbc.2023.104976 (PMC10374969; doi:10.1016/j.jbc.2023.104976)
Supplement: Supporting information [file mmc1.docx]

***The Journal of Biological Chemistry***

**Supplementary Information**

**SVCT2/SLC23A2 is a sodium-dependent urate transporter: functional properties and practical application**

Yu Toyoda^1,†^, Hiroshi Miyata^1,†^, Ryuichiro Shigesawa^1^, Hirotaka Matsuo^2^, Hiroshi Suzuki^1^, Tappei Takada^1,*^

1. Department of Pharmacy, The University of Tokyo Hospital, 7-3-1 Hongo, Bunkyo-ku, Tokyo 113-8655, Japan
2. Department of Integrative Physiology and Bio-Nano Medicine, National Defense Medical College, 3-2 Namiki, Tokorozawa, Saitama 359-8513, Japan

^†^ Yu Toyoda and Hiroshi Miyata contributed equally to this study.

^*^ **Correspondence to:**

Prof. Tappei Takada

Department of Pharmacy, The University of Tokyo Hospital, Tokyo, Japan; tappei-tky@g.ecc.u-tokyo.ac.jp

**Supplementary Figures S1 and S2**

**Supplementary Figures**

**Supplementary Figure S1. EGFP tag does not significantly affect the urate transport activity of human SVCT2.**

Using HEK293 cells transiently expressing SVCT2 (with or without EGFP-tag) 48 h after plasmid transfection, urate transport activities were examined in Krebs–Ringer buffer (pH 7.4) containing 10 μM [8-^14^C]-urate. Data are expressed as the mean ± SD; *n* = 3. ^**^, *p* < 0.01 vs. mock (Dunnett’s test).

**Supplementary Figure S2. EGFP tag does not significantly affect the urate transport activity of mouse Svct2.**

Using HEK293 cells transiently expressing Svct2 (with or without EGFP-tag) 48 h after plasmid transfection, urate transport activities were examined in Krebs–Ringer buffer (pH 7.4) containing 10 μM [8-^14^C]-urate. Data are expressed as the mean ± SD; *n* = 4. ^**^, *p* < 0.01 vs. mock (Dunnett’s test).
